# The Effects of Warming-Shifted Plant Phenology on Ecosystem Carbon Exchange Are Regulated by Precipitation in a Semi-Arid Grassland

**DOI:** 10.1371/journal.pone.0032088

**Published:** 2012-02-16

**Authors:** Jianyang Xia, Shiqiang Wan

**Affiliations:** 1 State Key Laboratory of Vegetation and Environmental Change, Institute of Botany, The Chinese Academy of Sciences, Xiangshan, Beijing, China; 2 Key Laboratory of Plant Stress Biology, College of Life Sciences, Henan University, Kaifeng, Henan, China; DOE Pacific Northwest National Laboratory, United States of America

## Abstract

**Background:**

The longer growing season under climate warming has served as a crucial mechanism for the enhancement of terrestrial carbon (C) sink over the past decades. A better understanding of this mechanism is critical for projection of changes in C cycling of terrestrial ecosystems.

**Methodology/Principal Findings:**

A 4-year field experiment with day and night warming was conducted to examine the responses of plant phenology and their influences on plant coverage and ecosystem C cycling in a temperate steppe in northern China. Greater phenological responses were observed under night than day warming. Both day and night warming prolonged the growing season by advancing phenology of early-blooming species but without changing that of late-blooming species. However, no warming response of vegetation coverage was found for any of the eight species. The variances in species-level coverage and ecosystem C fluxes under different treatments were positively dependent upon the accumulated precipitation within phenological duration but not the length of phenological duration.

**Conclusions/Significance:**

These plants' phenology is more sensitive to night than day warming, and the warming effects on ecosystem C exchange via shifting plant phenology could be mediated by precipitation patterns in semi-arid grasslands.

## Introduction

Climate warming has the potential to influence the structure and functioning of ecosystems [1], [2]. It can affect terrestrial primary production not only directly by changing plant photosynthesis [1] but also indirectly via extending the length of growing season [3]–[5], increasing soil nitrogen mineralization and availability [6], reducing soil water availability [7], and changing species composition [8], [9]. Given that these processes occur in different times at both seasonal (e.g., flowering in spring and senescence in autumn) and diurnal (e.g., photosynthesis during daytime and only plant respiration at night) scales, many uncertainties still remain unresolved in projection of the terrestrial carbon (C) feedback to climate warming.

At the seasonal scale, climate warming often leads to earlier flowering in spring and later senescence in autumn globally [10]–[12], indicating an extended period of active plant growth under warmer conditions. The extension of growing season may serve as one of the important mechanisms in enhancing ecosystem production under climate warming [10], [13]. For example, a growing body of results from atmospheric monitoring of carbon dioxide (CO_2_) and satellite remote-sensing of ecosystem production has revealed a positive dependence of net primary production (NPP) upon growing season length over the past decades [14]–[17]. However, the positive impact of prolonged growing season on NPP has been challenged in recent years because other processes under climate warming can counteract or reverse the positive impacts of warming-shifted plant phenology on ecosystem C uptake. For example, although premature flowering improves plant fitness, summer drought associated with climate warming can reduce reproductive success of plant species [18] and cancel out the C uptake of terrestrial ecosystem [19], [20]. In addition, the enhanced respiration by autumn warming can weaken the CO_2_ uptake enhancement induced by earlier growing season under spring warming [21]. Moreover, advanced budbreak under warming may lead to injury from a late-spring frost and longer leaf retention and increase the risk of freezing damage in the autumn [22]. All these studies suggest that the mechanism of warming effects on plant growth and terrestrial NPP is complex and the influences of lengthening growing season on ecosystem C sequestration may be regulated by other biotic and abiotic factors associated with climate warming.

At the diurnal scale, because plant photosynthesis occurs during daytime and there is only plant respiration at night, similar magnitudes of temperature increase during daytime and at night could bring differential impacts on ecosystem C cycling. Studies with numerous techniques, including manipulative experiments [23], [24], long-term observations [25], and model simulations [26], [27], have found differential influences of day vs. night warming on plant production. However, all the above findings have been attributed to changes in leaf-level C exchange processes under climate warming but neglected the influence of warming-shifted plant phenology on ecosystem C cycling. Only a few studies up to date have reported the impacts of day vs. night warming on plant phenology and their potential influences on ecosystem C exchange [28]. Historical meteorological records and model projections have revealed that climate warming occurs with greater magnitudes of temperature increase at night than during daytime [29]. Because the diurnal pattern of climate warming varies greatly among regions [30], understanding the possibly differential effects of day and night warming on plant phenology and their consequent influences on ecosystem C exchange will facilitate the projection of climate warming-terrestrial C feedback.

To address the issues raised above, we have conducted a field experiment to investigate the effects of day and night warming on phenology and ecosystem C exchange with four treatments, including control, day (06:00 am–06:00 pm, local time) warming, night (06:00 pm–06:00 am) warming, and whole-day warming in a semiarid temperate steppe in northern China since 2006. We experimentally tested the different effects between day and night warming on plant phenology, and explored the importance of plant phenology shifts in influencing ecosystem C exchange.

## Materials and Methods

### Study site and experimental design

This study was conducted in a semiarid temperate steppe in Duolun County (42°02′N, 116°17′E, 1324 m a.s.l.) in Inner Mongolia, China. Long-term (1953–2007) mean annual precipitation is approximately 383 mm with 90% of which falling from May to October. Mean annual temperature is 2.1°C with monthly mean temperature ranging from −17.5°C in January to 18.9°C in July. The sandy soil of the study site is classified as Haplic Calcisols according to the FAO classification, with bulk density of 1.31 g cm^−3^ and pH of 7.7.

The experiment has received the permits for the field study from the land owner, Institution of Botany, the Chinese Academy of Sciences. We used a complete random block design with 6 treatments, including control, day warming (06:00 a.m.–06:00 p.m., local time), night warming (06:00 p.m.–06:00 a.m.), whole-day warming (24 h), nitrogen addition, and whole-day warming plus nitrogen addition. We used control, day warming, night warming, and whole-day warming (e.g., ref. [24]) to examine the differential effects of day and night warming, and used control, nitrogen addition, whole-day warming, and nitrogen addition plus whole-day warming (e.g., ref. [31]) to test the interactive effects between nitrogen addition and whole-day warming. The effects of nitrogen addition and whole-day warming plus nitrogen addition were not included in the current study. Every treatment was replicated 6 times. With a 3 m distance between any two adjacent plots, thirty-six plots (3×4 m) were arranged in a 6×6 matrix. The warmed plots were heated using MSR-2420 infrared radiators (Kalglo Electronics Inc, Bethlehem, PA, USA) suspended at the height of 2.25 m above the ground. To simulate the shading effects of the infrared radiator, one “dummy” heater with the same shape and size as the infrared heater was suspended 2.25 m above ground in each unwarmed plot. The experimental plots were set up in September 2005 and warming treatments began on 23 April 2006. Because there are no living plants during late November to next early March and the warming effects on soil temperature during this period is very small (see Fig. 1 of [32]), the heaters were turn off during this period since the second year.

**Figure 1:**
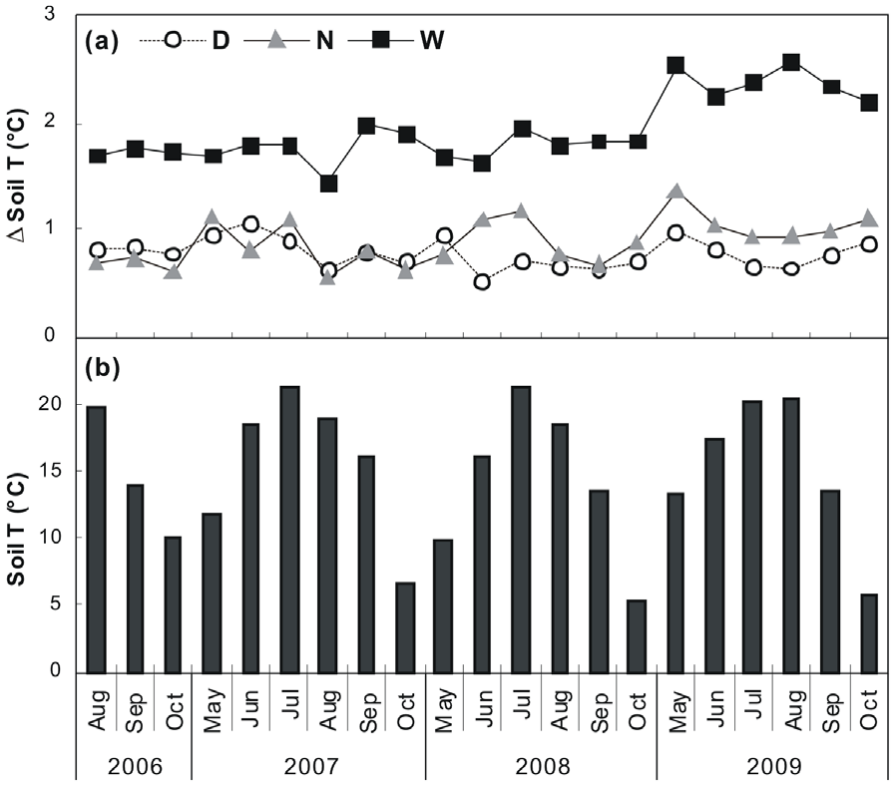
Monthly integrated values of (a) day (D), night (N), and whole-day (W) warming effects on soil temperature and (b) soil temperature in control plots. Soil temperature was measured at the depth of 10 cm.

Air temperature and precipitation used in this study were monitored hourly by a meteorological station (about 200 m away from the experimental plots) with an automatic system (Campbell Science Equipment, Logan, UT) at about 1.5 m above the ground.

### Measurements of soil temperature and moisture

Soil temperatures at the depth of 10 cm were recorded with an automatically Datalogger (STM-01 Soil Temperature Measurement System, Henan Electronic Institude, Zhengzhou, China). One temperature sensor was placed at the center in each plot. The data were stored at a 10-minute interval. Soil moisture at the depth of 10 cm was measured weekly using Diviner-2000 Portable Soil Moisture Probe (Sentek Pty Ltd., Balmain, Australia). Two measurements were taken weekly in each plot.

### Phenological observations

There were 50 plant species present in the experimental plot from 2006 to 2009. We monitored the flowering and fruiting phenology of 8 species over the entire growing season (from the earliest species, *Potentilla acaulis*, in May to the latest species, *Artemisia frigida*, in October) from 2006 to 2009. The 8 species include five forbs (*Potentilla acaulis*, *Potentilla bifurca*, *Potentilla tanacetifolia*, *Allium bidentatum*, and *Heteropappus altaicus*), two C_3_ grasses (*Agropyron cristatum* and *Stipa krylovii*), and one semi-shrub (*Artemisia frigida*). More information about the species traits and plant community structure can be found in a previous report in the same experimental site [9]. According to their natural phenological times, they could be divided into 3 groups including early (*P. acaulis* and *P. bifurca*), middle (*A. cristatum*, *P. tanacetifolia*, and *A. bidentatum*), and late (*S. krylovii*, *H. altaicus*, and *A. frigida*) species. The 8 species monitored in this study were dominant in the community at this site. Over the four growing seasons, the 8 plant species together accounted for 78% of the total aboveground biomass. Because these 8 species are the dominant species in the experimental plots, we only used their responses to represent the total community dynamics.

As soon as any of the 8 species produced obvious bud, we tagged five mature individuals for each species in each plot. The scoring of phenological stages was modified from Price and Waser [33], Dunne *et al.* [34], and Sherry *et al.* [3]. For forbs and semi-shrub, plant phenology was divided into 7 stages: plant not yet flowering (stage 0), unopened buds (stage 1), open flowers (stage 2), old flowers (post-anthesis; stage 3), initiated fruit (stage 4), expanding fruit (stage 5), and dehisced fruit (stage 6). For grasses, there were five stages: plant with flower stalks (stage 0), presence of spikelets (stage 1), exerted anthers and styles from the spikelet florets (stage 2), dried and broken-off anthers and styles (seed development; stage 3), and disarticulated seeds (stage 4). For grasses, we also scored the date when most culms in boot were visible as stage 1/2. Reproductive duration was calculated as the time between stage 1 (1/2 for grasses) and 5 (3 for grasses) for forbs and semi-shrub. Temporal overlap in the duration among different species was expressed as the time between the stage 1 (1/2 for grasses) of later species and the stage 5 (3 for grasses) of earlier species for forbs and semi-shrub. Therefore, we first calculated temporal overlap for each species and then summed them as the total temporal overlap in reproductive duration for all the 8 species.

For all species, each flowering stage present on every plant (whether in ray- or disk-florets) was noted. In each plot, we averaged all the stages present on the individuals to calculate a single weighted phenological score on each observation day. For forbs and semi-shrubs, the buds that did not open at the end of growing season were not included in the calculation of phenological score. For grasses, because some florets never developed seed, we assigned the stage after the presence of anthers as stage 3. The monitoring ended when all plants of a species have reached a phenological stage of 6 for forbs and semi-shrubs and 4 for grasses. If most of the fruits of a plant had dehisced and no more seed dehisced in the following 2 weeks, the data collections were ended.

### Coverage estimation

In August 2005, we established two permanent 1×1 m quadrats in each plot. Plant species composition was recorded in each quadrat at the end of August during the peak biomass by visually estimating percent cover of each plant species from 2006 to 2009. During the measurement, a 1×1 m frame with 100 equally distributed cells (10×10 cm) was put above the canopy in each quadrat. We first recorded the percent cover of each species in each grid, then summed all grids as total cover in each 1×1 m quadrats for each species, and at last used mean value of the two quadrats as the species percent coverage in each plot.

### Ecosystem C fluxes measurements

From May to October, ecosystem C fluxes were measured twice a month at 3-h intervals (8 times each measuring day; 06:00, 09:00, 12:00, 15:00, 18:00, 21:00, 00:00, and 03:00) with a transparent chamber (0.5×0.5×0.5 m) attached to an infrared gas analyzer (IRGA; LI-6400, LiCor, Lincoln, NE, USA). Two small fans ran continuously to mix the air inside the chamber. The polyethylene sheeting used for the chamber allows >90% of photosynthetic active radiation to pass into the chamber and the increases in air temperature during the measuring time period were less than 0.2°C. Two aluminum frames (0.5×0.5 m) were inserted 2–3 cm into the soil at two corners of each plot in 2005. During the measurement, the chamber was first placed and sealed on the frames for 20s and then CO_2_ concentrations were consecutively recorded during a 90-s period. We first measured net ecosystem C exchange (NEE), and then vented the chamber and replaced it on the frame and covered it with an opaque cloth. Because of elimination of light (and hence photosynthesis), the values of CO_2_ exchange represented ecosystem respiration (ER). Gross ecosystem exchange (GEE) was calculated as the difference between NEE and ER from 06:00 to 18:00. Seasonal net ecosystem productivity (NEP), ER, and gross ecosystem productivity (GEP) were calculated by multiplying daily integrated values of NEE, ER, and GEE, respectively, by the number of days since last measurement. Here NEP was defined as -ΣNEE, with negative value represents C source and positive value means C sink.

To measure total soil respiration, we inserted two PVC collars (11 cm in internal diameter and 5 cm in height; 2–3 cm into the soil) at two opposite corners in each plot. In order to exclude aboveground plant respiration, we removed all living plants inside the soil collars by hand at least one day prior to the measurements. Total soil respiration was measured by a LI-8100 portable soil CO_2_ fluxes system (Li-Cor, Inc., Lincon, NE, USA) twice a month with 3-h intervals (06:00, 09:00, 12:00, 15:00, 18:00, 21:00, 00:00, and 03:00). The values of total soil respiration used in the multiple analyses were calculated by multiplying daily integrated values.

### Data analysis

Because Richards equation is more flexible than the logistic equation to describe different shapes of growth data [35], we used Richard growth equation with the contraction-expansion algorithm [36] to fit phenological data from each species in each plot. The equation was described as:
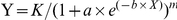
Where Y is the scored phenological stages (0–6 for forbs and for grasses 0–4). *K* is the maximum growth (here the last phenological stage, 6 for forbs and 4 for grasses); *a* is a parameter related to the first observation date; *b* is growth rate (phenological stage change per day) over time *X* (days since the first observation date); and *m* is a parameter related to the curve shape. First we estimate the 4 parameters by the contraction-expansion algorithm [36]. The method searches for optimal parameters by contracting and expanding search space alternatively with the objective of minimal residual sum squares. The parameter estimations were performed separately for each plot and species.

Times of the flowering (stage 2 for all species) and the fruiting (stage 3.5 for forbs and semi-shrub and 2.5 for grasses) were calculated by the calibrated Richards equation (see more details in supplemental text in ref [3]) for each species in each plot.

We used ANOVAs to test the warming effects on soil temperature and species coverage, and linear and multiple stepwise analyses (with *P*<0.10 as the criterion for selection) to examine the dependence of changes in species coverage and ecosystem C fluxes upon reproductive duration, accumulated precipitation and temperature, and temporal overlap among growing seasons. In each year, we summed the daily precipitation and air temperature within reproductive duration as the accumulated precipitation and temperature for different species. The total accumulated precipitation (or temperature) in a growing season was summed from accumulated precipitation (or temperature) of all species in that year. Repeated Measures ANOVA (RMANOVA) were used to examine the effects of species, day warming, and night warming on flowering time, fruiting time, reproductive duration, and coverage. RMANOVAs were also used to test the effects of day and night warming on species-level phenological events and ecosystem C fluxes. Between-subject effects were evaluated as day and night warming and within-subject effects were year. The species-level coverage from 2006 to 2009 used in the analysis was calibrated as minus the pretreatment data in 2005. The fitting of calibrated Richards equation was carried out in Matlab (Mathworks, Natick, MA) and all statistical analyses were conducted with SAS software (Version 8.01; SAS Institute Inc., Cary, NC, USA).

## Results

### Soil temperature

As expected, daily mean soil temperature at 10 cm depth was 0.77, 0.98, and 2.10°C higher in day, night, and whole-day warming treatment plots, respectively (Fig. 1a). The results of 3-way ANOVA showed both significant main effects of day and night warming (both *P*<0.001) on soil temperature. No interactive effect between year and day or night warming (both *P*>0.10) was detected, and there was no interaction between day and night warming on soil temperature (*P*>0.10) over the 4 growing seasons.

### Plant phenology

**Figure 2:**
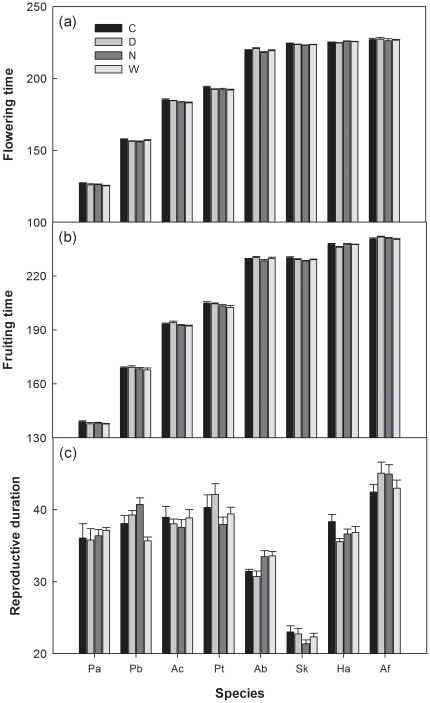
Species-level flowering time, fruiting time, and reproductive duration under control (C), day warming (D), night warming (N), and whole-day warming (W) treatments. Species are listed in the order of the mean time of buds first observed in the control plots over the four growing seasons, beginning in April with *P. acaulis* (Pa) and ending in October with *A. frigida* (Af). Inset panels represent the warming effects on phenological events of early (E), middle (M), and late (L) species. Data are mean ± SE for advanced (−) or delayed (+) phenology, respectively. *P. bifurca* (Pb), *A. cristatum* (Ac), *P. tanacetifolia* (Pt), *A. bidentatum* (Ab), *S. krylovii* (Sk), and *H. altaicus* (Ha).

From 2006 to 2009, the results of RMANOVAS showed that there were significant main effects of night warming, species, and year on both flowering and fruiting times (all *P*<0.01), whereas no effect of day warming on either flowering or fruiting time (both *P*>0.05) was detected (Fig. 2). Averaged over the 8 species, night warming advanced the flowering and fruiting time by 0.8 and 0.7 days, respectively (Fig. 2a, b; Supporting Information S1, S2). Neither day nor night warming (both *P*>0.10) affected reproductive duration over the 4 growing seasons (Fig. 2c; Supporting Information S3). No interactive effects of day × night warming or year × night warming were found on flowering time, fruiting time, or reproductive duration were observed (all *P*>0.10), whereas an interactive effect between year and day warming was detected on fruiting time (*P* = 0.010). Species did not interact with day or night warming to affect any of the phenology events (all *P*>0.05).

**Figure 3:**
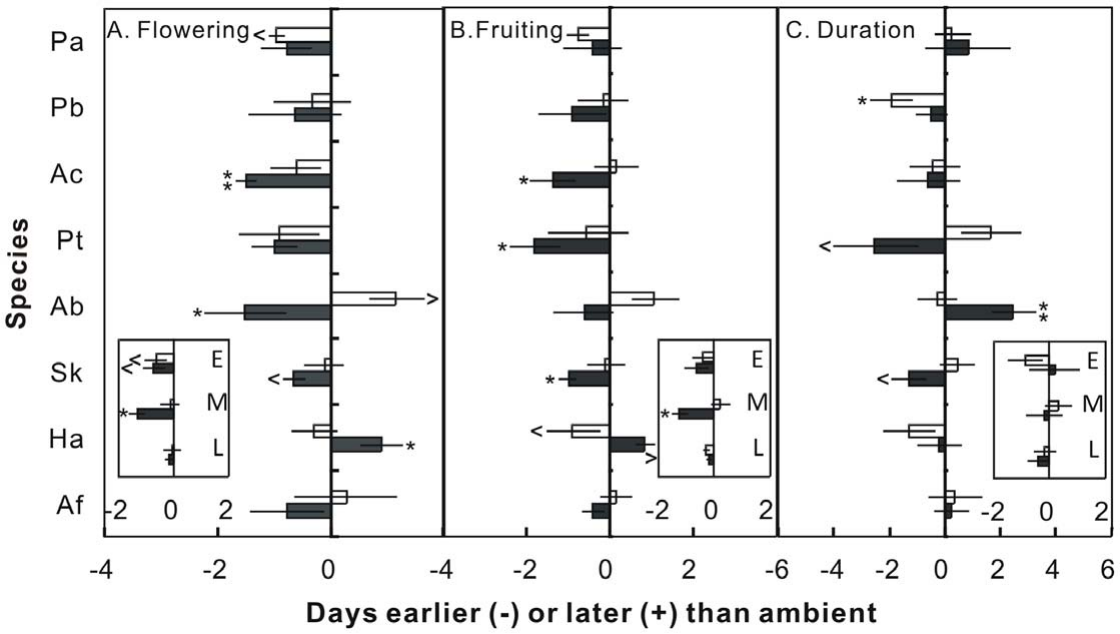
Changes in the flowering time (A), fruiting time (B), and reproductive duration (C) (in days) under day (open bars) and night (filled bars) warming. ∧, *P*<0.10; *, *P*<0.05, **, *P*<0.05. See Fig. 2 for species name abbreviations.

At the species level across the 4 growing seasons, day warming showed marginally significant effects on flowering times of *P. acaulis* (advanced by 1.0±0.1 days; *P* = 0.090) and *A. bidentatum* (advanced by 1.1±0.5 days; *P* = 0.080; Fig. 3A). Night warming accelerated the onset of flowering of *A. cristatum* (1.5±0.2 days; *P* = 0.009), *A. bidentatum* (1.5±0.7 days; *P* = 0.024), and *S. krylovii* (0.6±0.2 days; *P* = 0.083), while delayed the flowering time of *H. altaicus* (0.9±0.4 days; *P* = 0.042; Fig. 3A). Compared with flowering time, fruiting time was less responsive to elevated temperature. Day warming only marginally delayed the fruiting time of *H. altaicus* by 0.9 days (±0.6; *P* = 0.062). Night warming advanced the fruiting times of *A. cristatum*, *P. tanacetifolia*, and *S. krylovii* by 1.4 (±0.6; *P* = 0.048), 1.8 (±0.6; *P* = 0.033), and 1.0 (±0.2; *P* = 0.038) days, respectively (Fig. 3B), but marginally delayed the fruiting time of *H. altaicus* (0.9±0.2 days; *P* = 0.077; Fig. 3B). For reproductive duration, day warming only showed negative impacts on *P. bifurca* (−1.9±0.8 days; *P* = 0.032). Night warming significantly prolonged the reproductive duration of *A. bidentatum* (+2.5±0.8 days; *P* = 0.001) whereas shortened those of *P. tanacetifolia* (−2.5±1.5 days; *P* = 0.075) and *S. krylovii* (−1.3±0.6 days; *P* = 0.074; Fig. 3C).

When divided the 8 species into different phenological stages (early, middle, and late), both day (*P* = 0.097) and night (*P* = 0.067) warming showed marginally negative effects (or advancing effects) on flowering time of early species but neither of them (both *P*>0.10) influenced the flowering time of late species (Fig. 3a). The flowering time of middle species was significantly advanced by night (*P*<0.001) but not day (*P* = 0.683) warming (Fig. 3a). For the fruiting time, only night warming significantly advanced that of middle species (*P*<0.001; Fig. 3b). Neither day nor night warming impacted the reproductive duration of early, middle, or late species in this study (all *P*>0.10; Fig. 3c).

### Plant coverage and ecosystem C exchange

**Figure 4:**
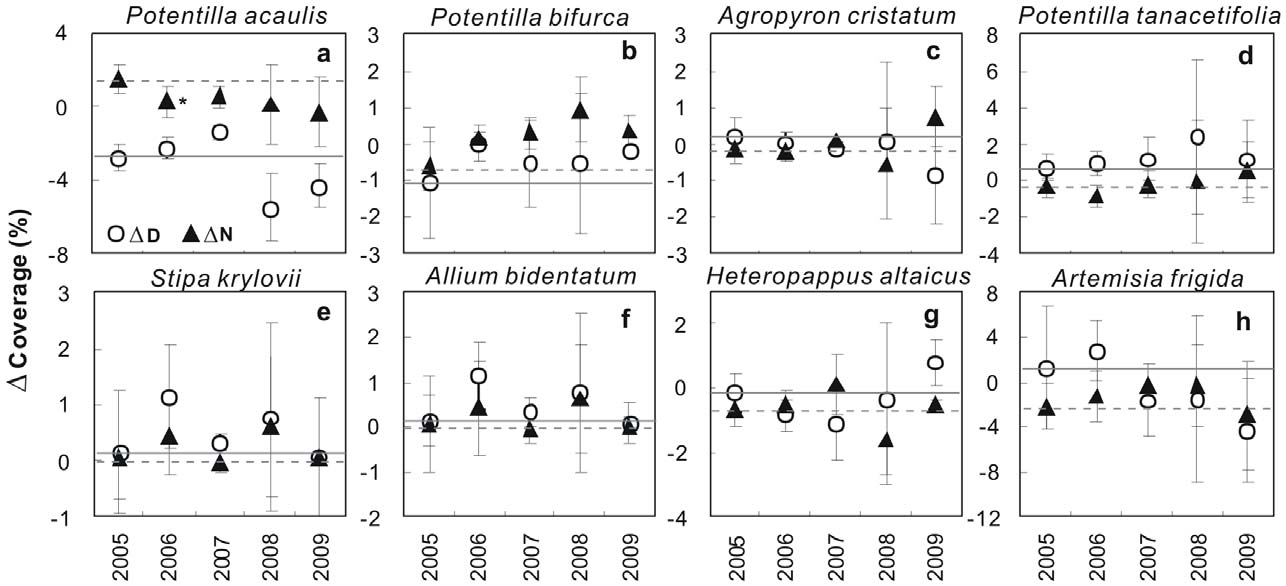
Coverage response of individual species to day (D) and night (N) warming from both pre-treatment (2005) and post-treatment (2006–2009). Pre-treatment (2005) values of day- and night-warming effects are indicated by horizontal gray solid and dashed lines, respectively. Means ± SE are shown. *, *P*<0.05. The y-axis is in different scales among the 8 species.

**Figure 5:**
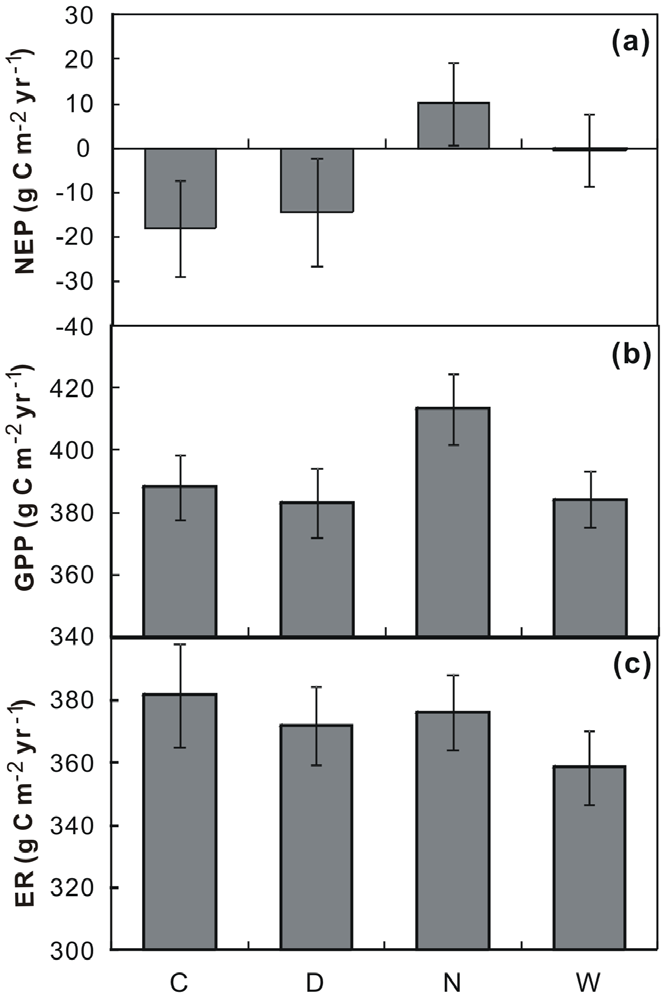
Mean values of net ecosystem productivity (a; NEP), gross primary productivity (b; GPP), and ecosystem respiration (c; ER) under control (C), day warming (D), night warming (N), and whole-day warming (W) from 2006 to 2009. Means ± SE are shown. Both the x- and y-axis are in different scales among the 8 species.

No response of species percent coverage to either day or night warming (both *P*>0.10) was found over the 8 species across the 4 growing seasons. When divided the data into different species, neither day nor night warming affected species-level coverage except for a marginally positive effect of day warming on the coverage of *P. bifurca* (*P* = 0.071; Fig. 4). In addition, the response directions of species coverage to warming treatments were opposite to the responses of reproductive duration for most of the 8 species (Fig. 3c and 4). Across the 4 growing seasons from 2006 to 2009, though RMANOVAs showed that neither GPP nor ER was significantly affected by day or night warming (all *P*>0.10), NEP (net ecosystem productivity) was not affected by day warming (*P*>0.10) but was significantly enhanced by night warming (*P* = 0.045; Fig. 5).

#### Biotic and abiotic factors influencing phenology, coverage, and ecosystem C exchange

**Figure 6:**
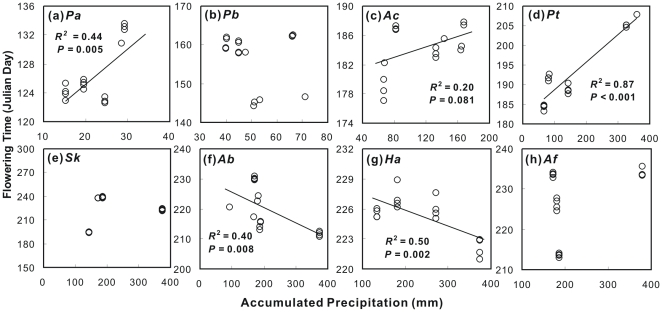
Relationships between the flowering time and accumulated precipitation in preceding periods (from Jan. 1) for the 8 species. See Fig. 2 for species name abbreviations.

Given the importance of water availability to plant growth in this ecosystem [9], we analyzed the relationship between the flowering time and the accumulated precipitation during the preceding period (from Jan. 1 to the date of flowering time) for each species. Across different treatments over the 4 years, positive dependence of flowering time upon precipitation were found for 3 spring-summer species (*P. acaulis*, *A. cristatum*, and *P. tanacetifolia*; Fig. 6a, c, d), while negative relationships were detected on 2 autumn species (*A. bidentatum* and *H. altaicus*; Fig. 6f, g).

When plant phenology was shifted, both biotic and abiotic factors which are important for plant growth and ecosystem C exchange would be changed. In this study, temporal overlap of reproductive duration among species was not affected by day warming but significantly reduced by night warming (*P*<0.001) over the 4 growing seasons, suggesting an increase in phenological complementarity under night warming. Though shifts in plant phenology induced changes in accumulated temperature and precipitation, no effect of either day or night warming on accumulated temperature within reproductive periods was found from 2006 to 2009 (*P*>0.10). Similarly, during the 4 growing seasons, accumulated precipitation within reproductive periods was not altered by either day or night warming except for a negative impact of night warming was detected in 2006 (*P* = 0.041). Across different treatments over the 4 growing seasons, stepwise multiple regression analyses showed that changes in accumulated precipitation within reproductive period explained 54.7% (*P* = 0.001), 61.4% (*P*<0.001), and 60.2% (*P*<0.001) of the variances in GEP, ER, and soil R, respectively (Table 1). Reproductive duration negatively affected GEP (partial *r* ^2^ = 0.018; *P* = 0.027), ER (partial *r* ^2^ = 0.053; *P*<0.001), and soil R (partial *r* ^2^ = 0.372; *P*<0.001) across the 4 growing seasons (Table 1). Accumulated temperature (partial *r* ^2^ = 0.523; *P* = 0.002) and species overlap (partial *r* ^2^ = 0.330; *P*<0.001) together explained 85.3% of the variations in NEP under different treatments across the 4 growing seasons (Table 1).

**Table 1:** Results of stepwise multiple regression analyses.

	Variable entered	Parameter estimate	Patial *r* ^2^	Probability
*GEP*	Accumulated P	5.449	0.547	0.001
	Accumulated T	1.480	0.062	0.002
	RD	−36.046	0.018	0.027
*ER*	Accumulated P	5.289	0.614	<.001
	RD	−45.534	0.053	0.000
	Accumulated T	1.415	0.025	0.063
*Soil R*	Accumulated P	4.815	0.602	<.001
	RD	−14.299	0.372	<.001
*NEP*	Accumulated T	0.953	0.523	0.002
	Species overlap	−3.060	0.330	0.000

Dependent variables: annual growing-season gross ecosystem productivity (GEP), ecosystem respiration (ER), total soil respiration (Soil R), and net ecosystem productivity (NEP); Independent variable: accumulated temperature (T) and precipitation (P), reproductive duration (RD), and temporal species overlap. Negative values of parameters estimates imply a negative relationships between the examined dependent variable and the independent variables.

**Figure 7:**
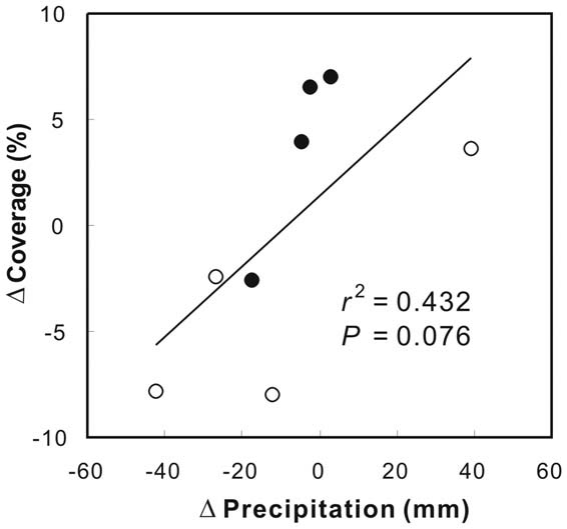
Dependence of warming-induced relative changes in the total plant coverage upon the changes in total accumulated precipitation over the 8 species within reproductive period from 2006 to 2009. Open circle, day warming; filled circle, night warming.

When the effects of day and night warming were pooled together, the warming-induced relative changes in the total coverage of the 8 species showed a positively linear dependence upon the changes in accumulated precipitation induced by shifts of reproductive periods across the 4 growing seasons (*r* ^2^ = 0.49, *P* = 0.076; Fig. 7). The results of stepwise multiple regression analyses (including the independent factors of reproductive duration, accumulated precipitation, accumulated temperature, and phenological overlap) showed that the changes in accumulated precipitation itself explained 48.3% (*P* = 0.056) variations of the changes in species percent coverage.

## Discussion

### Warming effects on plant phenology

It has widely been reported that earlier spring phenological events are associated with rising temperature under climate warming in recent decades [11]–[12], [37]–[39]. For instance, two previous meta-analyses [11]–[12] have summarized the globally coherent “fingerprint” of climate change and indicated that plant phenology has been already shifted in the past several decades. However, our result suggests that the response of plant phenology to climate warming is greatly dependent upon the diurnal pattern of increasing temperature. In this study, both flowering and fruiting times of the plant community were significantly advanced by night warming whereas neither of them was affected by day warming. The premature phenological stage under night warming in this study is consistent with the result from a previous experimental study across Europe which found night warming lead to earlier bud break for most species [40]. Given that the diurnal pattern of climate warming varies greatly among regions [30], the differential impacts of day and night warming on plant phenology could contribute to the large spatial variability in phenology shifts in the past decades [41], [42].

At the species level, both day and night warming showed various, including advancing, neutral, and delaying, effects on phenological times of the 8 species (Fig. 3). Our results are consistent with long-term observations of phenology from 385 British plant species, 16% of which flowered earlier whereas 3% flowered later in the 1990s compared with the previous 45 years [41]. This highlights diverse sensitivities and patterns of phenology among plant species in response to climate warming [40], [41], [43]. Although the responses among plant species differed substantially, both day and night warming advanced the flowering time of early blooming species, e.g. *P. acaulis*, but did not affect that of late-blooming species, leading to longer growing seasons (Fig. 3A and a). The extension of growing season under elevated temperature in this study is in accordance with the observations in many previous studies which used numerous techniques, including field observation [3], [44], [45], remote-sensing of ecosystem production [10], [46], monitoring of atmospheric CO_2_ concentration [15], and ecological modeling [17], [47]. Thus, at the community level, elevated temperature both during daytime and at night will prolong the growing season of the semi-arid grassland in northern China.

### Controlling factors of warming effects on ecosystem C exchange via shifting plant phenology

Although the length of growing season was extended, neither day nor night warming showed significant impact on species-level reproductive duration (Fig. 3C and c) or species percent coverage (Fig. 4) over the 4 growing seasons in this study. In addition, the warming effects on reproductive duration and coverage were in the opposite directions for most species (Fig. 3c and 4). At the ecosystem level, changes in reproductive duration showed negative influences on all GEP, ER and SR, which determine the NEP, under the different treatments across the 4 growing seasons (Table 1). The negative impacts of reproductive duration on ecosystem C exchange could be contributed to the greater temporal species overlap, which reduced phenological complementaity (thus enhanced the competition for resources) among species, at longer reproductive duration (Supporting Information S4). These results were not in agreement with those in previous studies that ecosystem production strongly depends upon the active growth length in the past several decades [17], [19], [48]. However, our results are consistent with a network study from 6 countries across Europe, in 5 of which observed insignificant response of biomass accumulation in the warmed plots irrespective of the extended length of growing seasons [40]. Similarly, no increase in alpine snowbed production in response to experimental lengthening of the growing season has recently been reported [49]. In addition, a 9-year eddy flux observation in a subalpine forest in the Colorado Rocky Mountains has found a negative relationship between net ecosystem production and growing season length [50]. Thus, although warming would prolong the growing season of plant community and change growth period of individual species, other factors may preclude or regulate the changes in biomass accumulation and ecosystem C exchange [40].

In this study, the accumulated precipitation within reproductive period was the predominant factor in regulating the variations in ecosystem C fluxes and vegetation coverage across all the treatments and growing seasons (Table 1), and the warming-induced changes in accumulated precipitation within reproductive periods was the main driver for the warming effects on species coverage (Fig. 7). These results indicate that water availability within plant active growth period will regulate the effect of warming-shifted plant phenology on ecosystem C exchange in this ecosystem. The results in this experiment are in accordance with some recent observations from larger scale studies. For example, a recent study in a subalpine forest found that longer growing season could reduce winter snow pack and thus soil water availability during summer, which will negatively affect ecosystem production [50]. Similarly, a previous study based on multi-year tower eddy flux measurement of CO_2_ exchange and phenology found that positive effects of earlier onset of flowering may be reduced by summer drought [19]. Up to now, only a few studies have been performed for the relationship between precipitation and plant phenology which found various effects of precipitation on times of phenological events. For example, Piao *et al.* [51] found that increased precipitation likely advanced the plant onset dates for temperate grassland. However, field experiments in northern America grasslands have not detected significant response of plant phenology itself to precipitation [3], [5]. The observations of climate and phenology in Europe have not revealed relationship between precipitation and phenology either [52]. In this study, we found different dependences of flowering time upon accumulated precipitation during the preceding period between spring-summer and autumn species (Fig. 6). A similar relationship between autumn phenology and accumulated precipitation during the preceding period has been reported for two autumn-flowering shrub species in Mediterranean area [53]. These results suggest the species-specific dependence of phenology on precipitation in this ecosystem. It is interesting that the negative effect of warming on soil moisture were greater in growing seasons with more total precipitation (Supporting Information S5). That means if plants move into wetter conditions, there will be a larger negative warming effect on plant growth via reducing soil moisture. Thus, precipitation patterns will critically mediate the role of warming-shifted plant phenology in regulating plant growth and ecosystem C exchange under the ongoing climate change.

### Differential impacts of day and night warming on ecosystem C exchange

From 2006 to 2009, neither reproductive duration nor accumulated precipitation within reproductive period was changed by day warming, which is consistent with the insignificant response of NEP under day warming with the data from the first 3 seasons (2006–2008) in this study [24]. The re-analysis of the 4-season data (2006–2009) in this study also found similar effect of day warming on NEP (Fig. 5a). Under night warming, NEP was enhanced over the 4 growing seasons though the accumulated precipitation within reproductive period was not changed. It suggests that other processes other than changes in water availability could be also important in mediating the different responses of ecosystem C exchange to day and night warming. In this system, two ecological processes may contribute to the positive response of NEP to night warming (Fig. 5a). One is phenological complementarity, which is an important mechanism by which competitive relationship of plant species affects ecosystem production [54]. In this study, night warming decreased temporal overlap and thus enhanced phenological complementaity and reduced the competition for limiting resources among species. Similarly, in an analysis of a long-term dataset, the reduction in flowering overlap among the plant species which shares pollinators also has been found in early-snowmelt years [55]. The increase in phenological complementarity will positively influence ecosystem production under night warming in this ecosystem. In fact, the multiple regression analyses showed that species temporal overlap was the dominant factor in influencing variations in NEP under different treatments across the 4 growing seasons (Table 1). The other reason could be leaf-level photosynthetic overcompensation, which has been found under night warming in this experiment [24]. Night warming has increased nighttime respiration and consumption of carbohydrates in leaves, and consequently stimulated plant photosynthesis and ecosystem C uptake in the subsequent days in this ecosystem [24], [56]. All the observations above indicate that both the effects of warming-shifted plant phenology and other associated ecological processes must be taken into consideration in predicting ecosystem C cycling under climate warming.

### Conclusions

This study has revealed that plant phenology in the temperate steppe in northern China was more sensitive to night than day warming. At the community level, both day and night warming caused longer growing seasons of plant community by advancing the onset of early-blooming species but unchanging that of late-blooming species. However, the impacts of warming-induced changes in the duration of active plant growth on vegetation coverage and ecosystem C exchange were mediated by the accumulated precipitation during phenological period. Although the accumulated precipitation within reproductive period was not altered by day or night warming in the experiments from 2006 to 2009, the regulation of precipitation on the warming effects on ecosystem C exchange can not be neglected as both the amount and temporal distributions of precipitation have been predicted to change in the future [28]. Our observations indicate that although climate warming will extend the length of growing season, its impact on ecosystem C exchange would not always be positive and could be mediated by precipitation patterns in semi-arid grassland. The differential effects of day and night warming on plant phenology highlight the importance of designing experimental studies with realistic warming trends in the future.

## Supporting Information

Supporting Information S1 Data of flowering time of all species and in all growing seasons.
(XLS)

Supporting Information S2 Data of fruiting time of all species and in all growing seasons.
(XLS)

Supporting Information S3 Data of reproductive duration of all species in all growing seasons.
(XLS)

Supporting Information S4 A figure showing the dependence of species overlaps on reproductive duration (RD) under different treatments across the 4 growing seasons.
(DOC)

Supporting Information S5 A figure showing the dependence of warming-induced changes in soil moisture of 0–10 cm upon total seasonal precipitation different treatments, including day warming (D), night warming (N), and whole-day warming (W), across the 4 growing seasons.
(DOC)
