# Supplementary material for: The Effects of Warming-Shifted Plant Phenology on Ecosystem Carbon Exchange Are Regulated by Precipitation in a Semi-Arid Grassland
Source: PLoS One. 2012 Feb 16;7(2):e32088. doi: 10.1371/journal.pone.0032088 (PMC3281109; doi:10.1371/journal.pone.0032088)
Supplement: Supporting Information S4 — A figure showing the dependence of species overlaps on reproductive duration (RD) under different treatments across the 4 growing seasons. (DOC) [file pone.0032088.s004.doc]

**Appendix S2**. Figure S1. Dependence of warming-induced changes in soil moisture of 0-10 cm upon total seasonal precipitation different treatments, including day warming (D), night warming (N), and diurnal warming (W), across the 4 growing seasons.
